# Supplementary material for: Microrobots powered by concentration polarization electrophoresis (CPEP)
Source: Nat Commun. 2023 Oct 6;14:6247. doi: 10.1038/s41467-023-41923-1 (PMC10558450; doi:10.1038/s41467-023-41923-1)
Supplement: Supplementary file 1 — Supplementary Information [file 41467_2023_41923_MOESM1_ESM.pdf]

Supplementary Information for
Microrobots Powered by Concentration Polarization Electrophoresis (CPEP)

Florian Katzmeier[1] and Friedrich C. Simmel[1*]

[1] *Department of Bioscience, TUM School of Natural Sciences, Technical University Munich, D-85748 Garching, Germany*

(Dated: September 19, 2023)

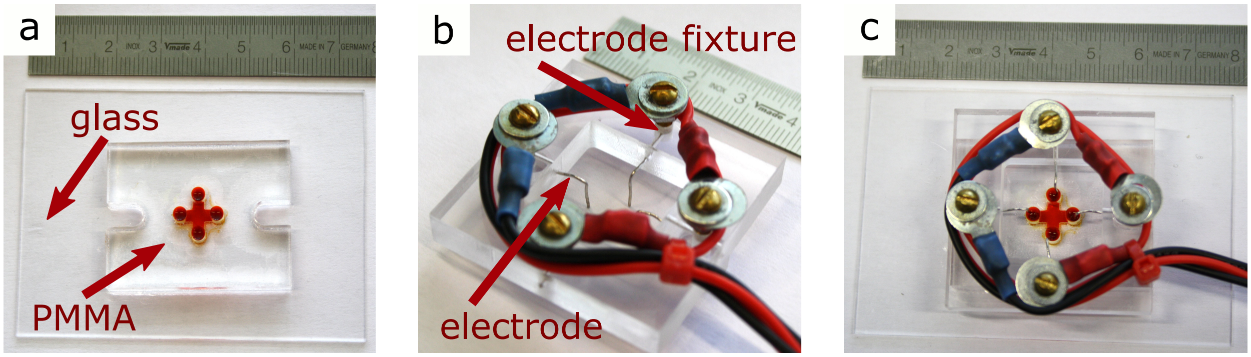

Supplementary Figure 1. Photographs of the experimental setup. (a) Sample chamber filled with a red dye. (b) Electrode mounting. (c) Electrode mounting placed on the sample chamber.

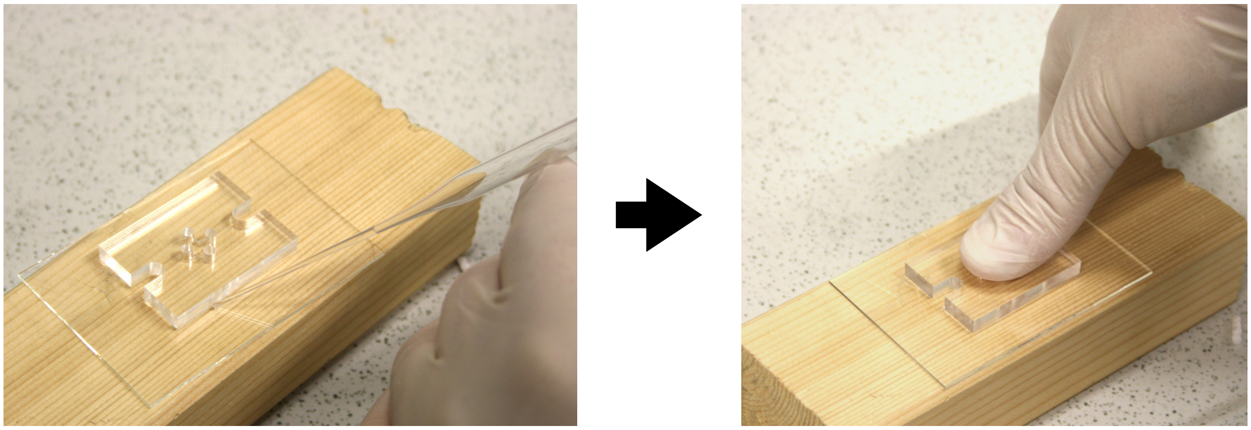

Supplementary Figure 2. Illustration of the glueing process used for manufacturing the sample chamber.

1. DESIGN OF THE EXPERIMENTAL SETUP AND ITS OPERATION

Photographs of our experimental setup are shown in Supplementary Figure 1. The sample chamber is shown in Supplementary Figure 1a, where it is filled with a red dye for better visualization. The sample chamber consists of two parts. Its bottom is constituted by a glass cover slide, whereas its top is made from a piece of PMMA, from which the channel geometry has been milled out via micro-milling. Both parts are glued together with dichloromethane. The gluing process is illustrated in Supplementary Figure 2. First, the PMMA part is placed on the glass cover slide. Then small drops of dichloromethane are placed with a glass pipette at the edge of the PMMA part. Due to capillary forces, the drops wet the surface between the PMMA and the glass

* Corresponding author - email: simmel@tum.de

slide. The dichloromethane dissolves the surface of the PMMA. The PMMA part is then pressed gently with a finger onto the glass cover slide until the dichloromethane is dried. Supplementary Figure 1b and Supplementary Figure 1c show the electrode mounting, where four electrodes are fixed on a PMMA frame. The electrodes extend out of small holes from the PMMA frame and are clamped with a screw from above (see red arrow in Supplementary Figure 1b with the label “electrode fixture”). In Supplementary Figure 2c the electrode mounting is placed on the sample chamber such that the electrodes extend into the inlets of the sample chamber, resulting in an operational setup.

2. CHARACTERISTIC FREQUENCIES AND VELOCITY SCALES

In this section, we compute the characteristic frequencies and magnitudes of the slip velocity for CPEO flows around charge dielectric particles and for ICEO flow around dielectric and metallic particles. We use the average particle radius $R = 0.78\,\mu\text{m}$ of the two particles composing our dimers as the typical size. Further, we use the inverse averaged diffusion constant as used by [1] and [2] for the diffusion constant which is defined by $D = \frac{2D^+D^-}{D^++D^-}$. D^+ and D^- are the ion diffusion constants for $TrisH^+$ and Cl^-, respectively, and are given by $D^+ = 800\,\mu\text{m}^2/\text{s}$ and $D^- = 2000\,\mu\text{m}^2/\text{s}$. Numerically, $D = 1143\,\mu\text{m}^2/\text{s}$ for this case.

The characteristic frequency of ICEO flows around metal particles is derived from the charging time $\tau = \lambda R/D$[3] of the electric double layer on the particle surface, where λ is the Debye screening length and depends on the ionic strength c. A concentration of $c = 50\,\mu\text{M}$ corresponds to $\lambda = 43\,\text{nm}$. The characteristic frequency is then given by

$$f_c = \frac{1}{2\pi}\frac{D}{\lambda R}, \tag{1}$$

which results in $f_c = 5396$ Hz.

For ICEO flows around dielectric particles the characteristic time scale is given by $\tau = \lambda^2/D$ [3] which corresponds to a characteristic frequency of

$$f_c = \frac{1}{2\pi}\frac{D}{\lambda^2}, \tag{2}$$

which gives $f_c = 98.0\,\text{kHz}$.

Note that the equation for the characteristic time scale for dielectric particles in [3] has an additional erroneous factor ϵ_w/ϵ_d (see their equation 6.14). ϵ_w/ϵ_d is the fraction of the dielectric

constants of water and the particle. The equation above can be obtained when starting from their equation 6.7 and by following the steps stated in the paper. Additionally, one has to assume $\frac{1+\epsilon_w/\epsilon_d}{\epsilon_w/\epsilon_d} \approx 1$, which is reasonable as in most cases $\epsilon_w \gg \epsilon_d$.

The characteristic time scale for CPEO flows is independent of the Debye screening length and is given by $\tau = R^2/D$ [1]. Here, τ is the typical time that ions need to diffuse across the length of our dimer. The characteristic frequency is given by

$$f_c = \frac{1}{2\pi}\frac{D}{R^2}, \tag{3}$$

which for our parameter settings is $f_c = 297\,\text{Hz}$.

The slip velocity V for ICEO flows around a metal particle is given by

$$V = \epsilon_w \frac{RE^2}{\mu} \tag{4}$$

where ϵ_w is the dielectric constant of water and μ is the viscosity. When using the maximum applied electric field amplitude $E = 16.75\,\text{mV}/\mu\text{m}$, we obtain $V = 0.33\,\mu\text{m/s}$.

The slip velocity V for ICEO flows around a dielectric particles is given by

$$V = \frac{3}{4}\epsilon_d \frac{\lambda E^2}{\mu} \tag{5}$$

where ϵ_d is the dielectric constant of the particle [4]. We used the relative permittivity of 3.7 for silica to compute ϵ_d. When again using the maximum applied electric field amplitude we get $V = 0.33\,\mu\text{m/s}$.

The slip velocity V for CPEO flows around a charged dielectric particle is given by

$$V = U\epsilon_w \frac{RE^2}{\mu} \tag{6}$$

where U is the dimensionless velocity computed which has typically a magnitude of $U = 0.1$ (see below and in [1]). For the maximum applied electric field amplitude this results in $V = 17.47\,\mu\text{m/s}$.

3. PROPULSION DIRECTION

In this section, we further investigate the observation that the migration direction of the microswimmers is influenced by the buffer composition, specifically by the presence or absence of $MgCl_2$. The observed change in the migration direction could be attributed either to a

complete inversion of the hydrodynamic flow or to a different curvature of the flow lines. The results obtained by Fernandez Mateo et al. [1], who computed the magnitude U of CPEO flows around spherical particles in AC-electric fields, show that flow reversal is theoretically feasible. However, this reversal occurs under physical conditions that are difficult to achieve, such as exceedingly high zeta potentials ($\zeta > 100\,\mathrm{mV}$) and unrealistically low ionic diffusion coefficients ($D \approx 500\,\mu\mathrm{m}^2/\mathrm{s}$). We therefore used tracer particles to experimentally investigate the flow field for both migration directions. The flow fields derived from these experiments indicate that the flow field around the dimer is merely differently curved for the different conditions, but not reversed. Finally, we studied the impact of the DNA modification and $MgCl_2$ on the surface properties of our microswimmers by measuring the electrophoretic mobility of the monomer particles via electrophoretic light scattering. We found that the DNA modification did not alter the monomer mobility which suggests that the inversion of the migration direction is in fact not related to the DNA modification. Our results indicate that small amounts (5 µM) of $MgCl_2$ significantly alter the monomer mobility but do not change the surface charge. Since Fernandez Mateo et al.'s result for the flow velocity only applies to monovalent ions with identical diffusion constants, the significant effect of even small amounts of divalent magnesium ions on the mobility lets us to surmise that a more general theory for the dimensionless flow velocity might elucidate the reasons for the observed migration direction reversal.

3.1. Flow reversal

In this section, we plot the result of [1] for the dimensionless flow magnitude U of CPEO flows around spherical particles for different ion diffusion constants. Their result for U is a function of the zeta potential ζ, the Dukhin number Du, and the ion diffusion constant D. The Dukhin number is a dimensionless quantity that characterizes the surface conduction of colloidal particles. In Supplementary Figure 3, we plot the dimensionless flow magnitude U versus the dimensionless frequency $2\pi f/(D/R^2)$ for different ion diffusion constants D and Dukhin numbers Du at a constant zeta potential $\zeta = 101\,\mathrm{mV}$. The plot on the upper left is a recreation of a plot shown in [1], where we plotted the frequency response of U for the ion diffusion constants of KCl ($D = 2036\,\mu\mathrm{m}^2/\mathrm{s}$) and four different values of the Dukhin number. In the other plots, we decreased the diffusion constant. We find decreasing velocities for the higher Dukhin numbers, 1 and 10, for decreasing values of the ion diffusion constant. For very low diffusion constants

($D = 226\,\mu\mathrm{m}^2/\mathrm{s}$) and Dukhin numbers of 1 and 10, we find flow reversal at low frequencies.

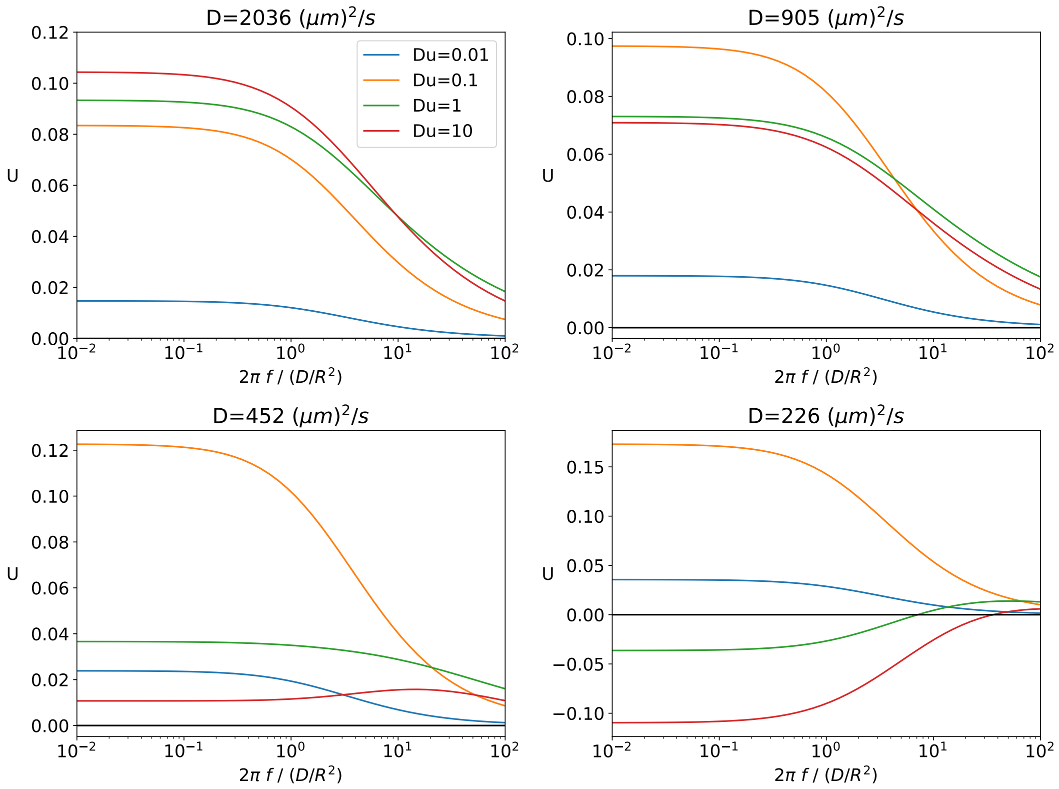

Supplementary Figure 3. Plots of the frequency response of the dimensionless flow magnitude of CPEO flows around spheres for different diffusion constants D and Dukhin numbers Du. The dimensionless zeta potential was set to $\zeta = -4$. The upper left plot is a recreation of the plot shown in [1]

In Supplementary Figure 4, we systematically plotted the frequency response of the flow magnitude for different values of the surface charge density σ. Within one graph, we plotted the frequency response for several values of the ion diffusion constant. Note that the zeta potential ζ is a function of the surface charge density and the Debye length λ, which is in turn a function of the salt concentration c that we set to $\lambda = 43\,\mathrm{nm}$ and $c = 50\,\mu\mathrm{M}$. We assume a particle radius of $R = 0.78\,\mu\mathrm{m}$. We vary the surface charge since both the Dukhin number and the zeta potential are functions of the surface charge. For low surface charges ($\sigma = -0.0005\,\mathrm{C/m^2}$, $\sigma = -0.0010\,\mathrm{C/m^2}$, $\sigma = -0.0015\,\mathrm{C/m^2}$) and low zeta potentials, we find

overall increasing flow magnitudes U for increasing surface charges. However, at high surface charges ($\sigma = -0.0045\,\text{C/m}^2$, $\sigma = -0.0080\,\text{C/m}^2$, $\sigma = -0.0160\,\text{C/m}^2$), we find decreasing flow magnitudes for increasing surface charges, which decrease more strongly for lower ion diffusion constants. This leads, at a certain point, to a flow reversal at low frequencies. Interestingly, the graphs cross the zero line at higher frequencies and become positive again. Overall, we find that flow reversal can indeed occur for high surface charges and low diffusion constants. However, the required values for the surface charges/zeta potentials ($\zeta > 100\,\text{mV}$) and the diffusion constants ($D \approx 500\,\mu\text{m}^2/\text{s}$) are physically rather unrealistic.

3.2. Hydrodynamic flow field

In order to gain further insight into the reversal of migration direction, we experimentally visualized the hydrodynamic flow which propels our microswimmers (cf. Fig. 1e of the main text). To this end, we recorded brightfield videos at a 60x magnification, capturing a migrating microswimmer surrounded by smaller spherical particles serving as tracers. We specifically studied conditions under which the microswimmer either migrated forwards ($25\,\mu$M NaOH and $5\,\mu$M $MgCl_2$) or backwards ($50\,\mu$M NaOH and $0\,\mu$M $MgCl_2$).

As tracer particles we used unmodified silica spheres from Microparticles GmbH, with a diameter of $0.70\,\mu$m (Lot: SiO_2–COOH-F-SC143-10ml). We selected silica because of its high density, which ensures that these small particles do not quickly diffuse out of the focal plane. The size of these particles was chosen as a compromise as they need to be small enough to not disturb the flow field, yet large enough to make tracking feasible.

We applied an electric field with an amplitude of $16.75\,\text{mV}/\mu\text{m}$ and a frequency of $750\,\text{Hz}$. This frequency was higher than in our usual experiments as we were imaging the microswimmer and tracer particles at a higher magnification, where electrophoretic oscillations were visible at $f = 250\,\text{Hz}$. Our samples were prepared similarly to our buffer screening experiments, with the washing protocol for the tracer particles mirroring that of our DNA-modified colloids, substituting buffer with water in all washing steps.

During the recording process, we manually adjusted the microscopy stage in sync with the migrating microswimmer to maintain it within our field of view. Due to the imperfect manual position adjustment, we employed an ImageJ plugin, Template Matching and Slice Alignment [5], to obtain microscopy videos with a stationary microswimmer and moving tracer particles. The re-

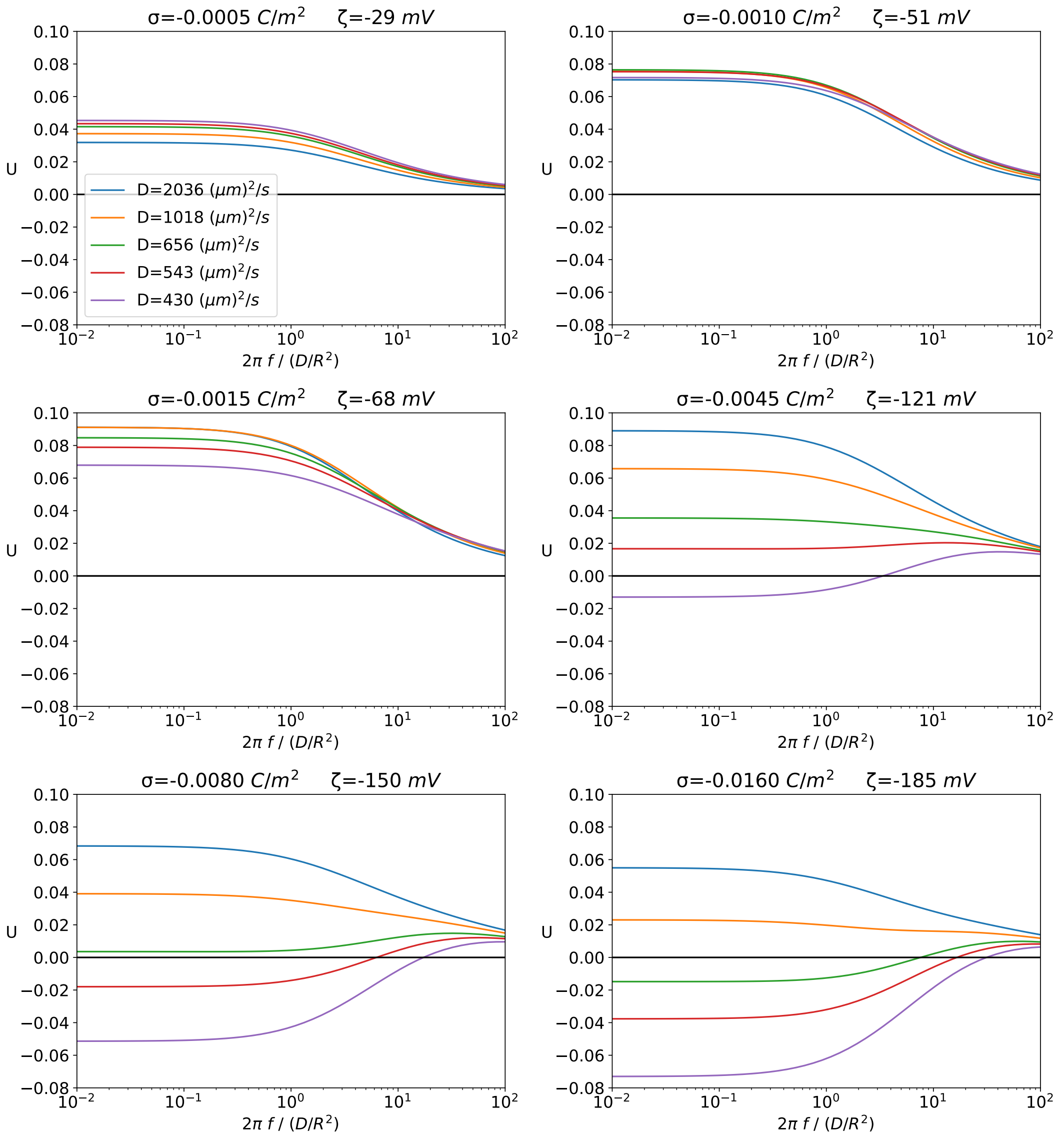

Supplementary Figure 4. Plots of the frequency response of the dimensionless flow magnitude for CPEO flows around spheres with different surface charges σ and diffusion constants D. The Debye length is set to $\lambda = 43\,\mathrm{nm}$ and the particle radius is $R = 0.78\,\mu\mathrm{m}$

sulting videos are provided in the Supplementary Movie files 3 and 4.

In Supplementary Figure 5a and Supplementary Figure 5b, we visualized the trajectories of tracer

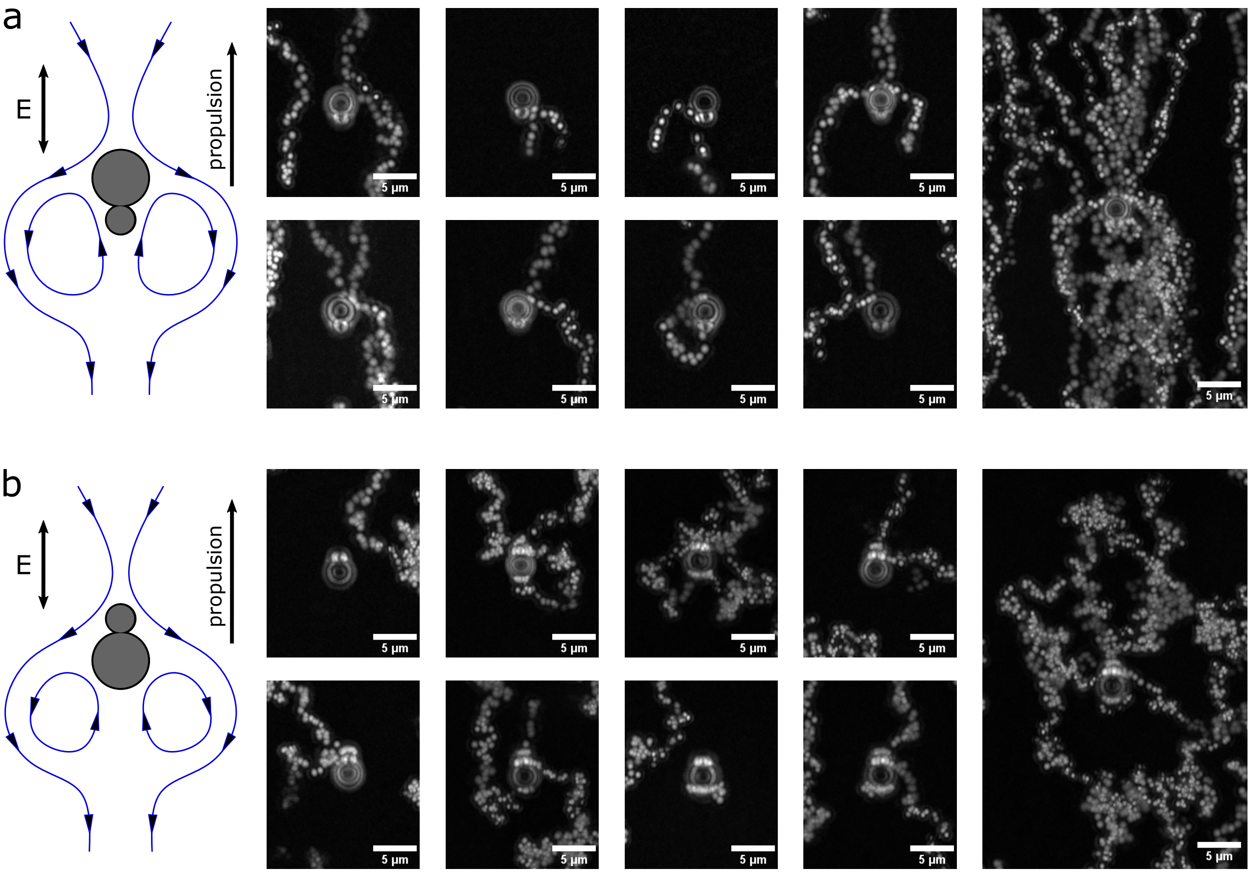

Supplementary Figure 5. Overlay images showing particle traces of silica spheres, drifting in the flow field created by the microswimmer. Part (a) of the figure displays a forward-moving microswimmer with the larger particle located in the front, and part (b) displays a backward-moving microswimmer. The electric field oscillates in the vertical direction. The sketches on the left depict the streamlines of the deduced hydrodynamic flow, wherein the tracer particles drift. The smaller images in the center display short overlays involving one or two tracer particles, whereas the larger images on the right show an overlay from a longer video, capturing a larger number of tracer particles. The videos are provided in the Supplementary Movie files 3 and 4.

particles for the forward and backward migrating microswimmer, respectively. For this purpose, we selected segments of the recorded video that contained at most two tracer particles passing by the microswimmer. From the individual frames F_i of the selected video segments, we computed $G_i = \left|F_i - \overline{F}\right|$ where $\overline{F}$ is the average image of the F_i. In the images G_i, particles appear as bright spots, independent of their appearance in the images F_i where they can appear either bright or dark depending on their position relative to the focal plane.

We then created an overlay image by extracting the maximum value of each pixel in the image sequence from the images G_i. The overlay images for both migration directions are shown on the right side of Supplementary Figure 5. On the left side of Supplementary Figure 5, we have sketched the flow field in which the tracer particles drift that can be deduced from the images. On the right, there is an overlay image of a longer video segment containing many tracer particles.

For the forward moving microswimmers in Supplementary Figure 5a, particles approach the microswimmer from the front and move towards the center of the larger particle. They are then expelled perpendicularly to the migration direction of the microswimmer. Occasionally, a particle is trapped behind the microswimmer, where it circulates in the flow field. These trajectories are representative of the fluid flow in Fig. 1 of the main text. Importantly, it should be noted that hydrodynamics is not the only factor influencing the movement of the tracer particles. Induced dipole-dipole forces also contribute to the interactions between the microswimmer and the tracer particles.

For the backward moving microswimmers in Supplementary Figure 5b, the trajectories are less clear as the overall flow magnitudes are found to be lower. The trajectories appear more diffuse since the Brownian motion of the tracer particles dominates over their drift in the microswimmer's flow field. Despite this, tracer particles are observed to approach the microswimmer from the front, now represented by the smaller particle. They are attracted to the center of the smaller particle and then expelled perpendicular to the microswimmer's migration direction. Occasionally, a particle that has already passed by the microswimmer is attracted to its rear, i.e., the larger particle of the microswimmer. The tracer particle then becomes attached to the larger particle, which we attribute to induced dipole-dipole forces. We hypothesize that the induced dipole-dipole forces are stronger compared to the hydrodynamic flow in this scenario, preventing the circulation that is observed for the forward-moving microswimmer. Nonetheless, we surmise that the flow field still corresponds to the sketch on the left of Supplementary Figure 5b.

We conclude that the flow field, is curved differently for backward-migrating microswimmers compared to forward-migrating microswimmers, and it is not simply inverted.

3.3. Monomer mobility

To further investigate whether the inversion of migration direction is associated with the DNA functionalization of the colloids, we measured the mobility of the $1.0\,\mu$m-sized silica particles, both with and without DNA modification, using electrophoretic light scattering. Using a Malvern Zetasizer Nano ZS with a Dip-Cell accessory, we tested our samples in a single-use cuvette (Einmalküvetten ROTILABO® PMMA, Macro, 4 ml) from Carl Roth. We assessed the monomer mobility under buffer conditions that induced both forward and backward microswimmer migration. As we were interested specifically in the effect of small quantities of $MgCl_2$ ($5\,\mu$M), we carefully implemented several washing steps to avoid salt contamination. To this end, the unmodified $1.0\,\mu$m silica particles were diluted 1 to 5 in water resulting in concentrations comparable to those of our modified silica particles. The unmodified particles were washed following the same procedure as that used for the DNA-coated particles, but the borate buffer washing steps were omitted. For all measurements, we used the same cuvette, which was rinsed with deionized water between runs. The Dip-Cell was also rinsed between measurements. We marked one side of the cuvette to maintain the same orientation across all runs. Sample volumes of 1 mL were used, and colloids were diluted by a factor of 1 to 1000, i.e., we used $1\,\mu$L of our stock solution. The buffer was mixed directly in the cuvette for each measurement. We conducted four measurements, with 30 runs for each pipetted sample at a temperature of $25\,^{\circ}$C. The corresponding measurement files and raw data are provided as a Source Data file. To mitigate systematic errors, we pipetted each sample for each buffer condition three times, resulting in a total of 12 values for the monomer mobility under each buffer condition. We computed averages and standard deviations from these, which are represented in Supplementary Figure 6 as bar graphs and error bars for the different buffer conditions used ($25\,\mu$M and $50\,\mu$M of NaOH, both with and without $5\,\mu$M of $MgCl_2$, and also $MgCl_2$ alone at $5\,\mu$M).

We did not observe any significant variation between the mobility of DNA-functionalized and unmodified colloids within the computed standard deviations, suggesting a negligible impact of the DNA modification on the electrokinetic properties of the colloids, even though the mobility of the DNA-modified colloids is lower than the mobility of the unmodified colloids across all buffer conditions. Furthermore, we found that the presence of $5\,\mu$M of $MgCl_2$ significantly reduced the mobility compared to experiments performed at the same NaOH concentrations without $MgCl_2$. We estimated the surface charge of the monomers using the measured mobility values and the

buffer conditions of our experiments. This analysis was undertaken to examine whether the presence of $MgCl_2$ only modifies the double layer structure through enhanced electrostatic screening, or whether it also changes the surface charge. The zeta potential ζ can be estimated from the mobility μ using the Smoluchowski equation: $\zeta = \mu \frac{\eta}{\varepsilon_w \varepsilon_0}$ Here, η represents the viscosity of the solution, while ε_w and ε_0 denote the relative permittivity of water and the vacuum permittivity, respectively. From the zeta potential, we computed the surface charge with the help of the Poisson-Boltzmann equation, which in the presence of four ion species is given by:

$$\varepsilon_w \varepsilon_0 \frac{d^2\Phi}{dx^2} = -\left[+e \cdot c_{\text{NaOH}} \cdot e^{\frac{-e\zeta}{k_B T}} - e \cdot c_{\text{NaOH}} \cdot e^{\frac{e\zeta}{k_B T}} + 2e \cdot c_{\text{MgCl}_2} \cdot e^{\frac{-2e\zeta}{k_B T}} - 2e \cdot c_{\text{MgCl}_2} \cdot e^{\frac{e\zeta}{k_B T}}\right] \tag{7}$$

Here, e is the elementary charge, k_B is the Boltzmann constant, and T is the absolute temperature of the solution. c_{NaOH} and c_{MgCl_2} represent the concentrations of NaOH and $MgCl_2$ in the solution, respectively. Similar to the two ion species case, we can derive a relation between the surface charge σ, the charge per unit area on the surface of the monomers, and the zeta potential ζ. By multiplying the Poisson-Boltzmann equation with $\frac{d\Phi}{dx}$, integrating the result, and applying the boundary conditions $\phi(\infty) = 0$ and $\frac{d\Phi}{dx}(\infty) = 0$ and at the surface, $\phi(0) = \zeta$ and $\frac{d\Phi}{dx}(0) = -\frac{\sigma}{\varepsilon_w \varepsilon_0}$, we obtain the following equation for σ:

$$\begin{aligned} \sigma &= -\sqrt{2\varepsilon_w \varepsilon_0 k_B T \cdot Q} \\ Q &= c_{\text{NaOH}} \left(e^{-e\zeta/k_B T} - 1\right) \\ &\quad + c_{\text{NaOH}} \left(e^{e\zeta/k_B T} - 1\right) \\ &\quad + c_{\text{MgCl}_2} \left(e^{-2e\zeta/k_B T} - 1\right) \\ &\quad + 2c_{\text{MgCl}_2} \left(e^{e\zeta/k_B T} - 1\right) \end{aligned}$$

Based on this equation, we computed the surface charge from the measured mobilities for our experimental buffer conditions. We also accounted for the standard deviation of the zeta potentials, $\delta\zeta$, by using error propagation $\delta\sigma = \left|\frac{\partial\sigma}{\partial\zeta}\right| \cdot \delta\zeta$, which allowed us to determine the standard deviation of the surface charge, $\delta\sigma$.

The results are shown as a bar graph in Supplementary Figure 7, indicating that the surface charge remains the same for identical NaOH concentrations regardless of the addition of 5 μM of $MgCl_2$. Moreover, we observed an increase in surface charge as the NaOH concentration increased. This trend is expected, as higher NaOH concentrations result in a greater degree of deprotonation of the carboxyl groups on the colloidal surface. Given the pronounced effect of

even minor quantities of divalent magnesium ions on the mobility, we surmise that a detailed mathematical treatment of the dimensionless flow velocity in the context of mixed electrolytes, including divalent ions, could provide better insight into the observed reversal of migration direction.

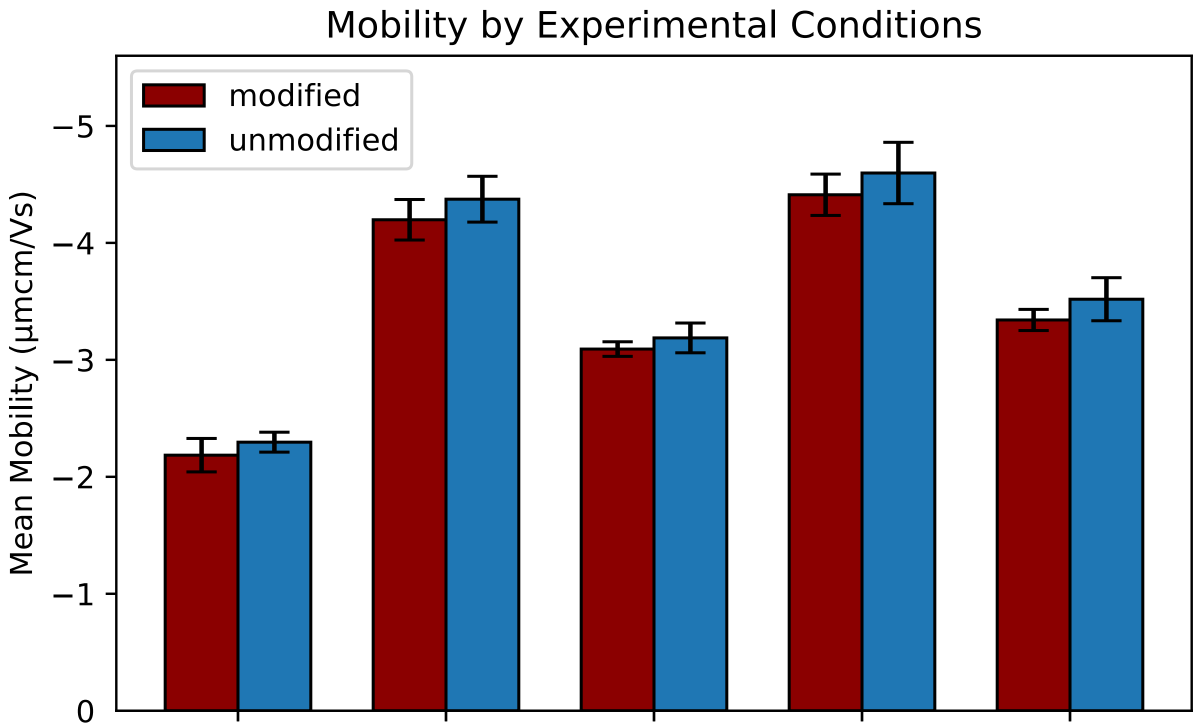

$MgCl_2$ (μM)	5	0	5	0	5
NaOH (μM)	0	25	25	50	50

Supplementary Figure 6. Bar graphs of the average monomer mobility from four measurements at each buffer condition. The error bars indicate the standard deviation. The buffer conditions are listed in the table below. Mobilities of DNA-modified and unmodified colloids are plotted next to each other in red and blue, respectively. No significant difference was observed between the mobility of DNA-modified and unmodified colloids across all buffer conditions. Source data are provided as a Source Data file.

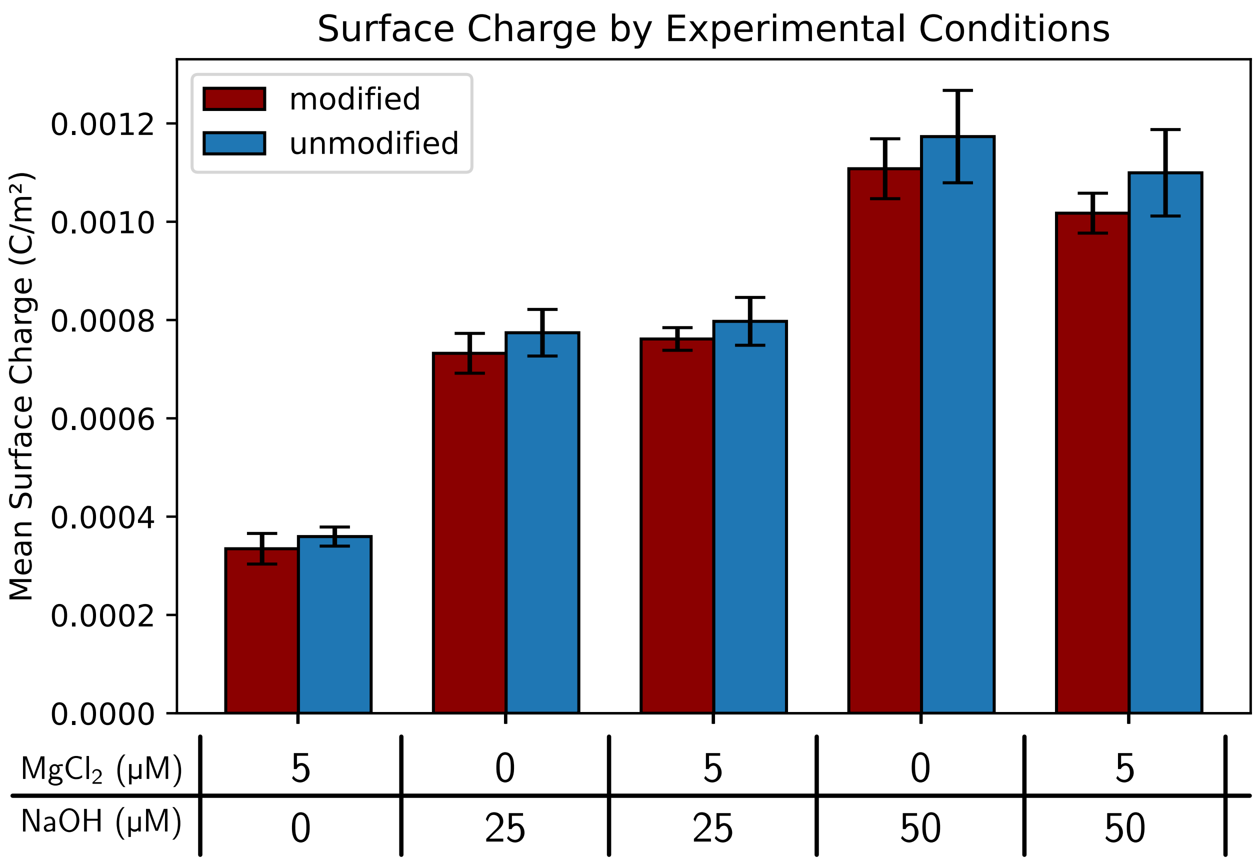

Supplementary Figure 7. Bar graphs depicting the surface charge of monomers calculated from the average monomer mobility at each buffer conditions. The error bars are derived from the standard deviation of the mobilities via error propagation. The surface charges of DNA-modified and unmodified colloids are plotted next to each other in red and blue, respectively. The surface charge increases with higher concentrations of NaOH and is unaffected by the presence of $MgCl_2$.

4. FUNCTIONALIZATION OF THE COLLOIDS

4.1. DNA sequences

The sequences of the oligonucleotides used for modification and cross-linking of the microparticles (in 5' to 3' direction) are listed below:

- `/5AmMC6/TTCGTTTTAGTCCCATTTGTTCAGTTTTTTCAGTTTTAGCGCGGTAGTTCAGTTAGGTCA`
- `/5AmMC6/GTCTTTTATGCTGCTTATTCGTGTATATCCTGACCTAACTGAACTACCGCGCTAAAACTG`

Here '/5AmMC6/' denotes the amino modification at the 5' end of the sequences by the oligo synthesis company (IDT).

4.2. Buffer and reagent stocks

The following buffers and reagents can be prepared in advance.

- $1\,\mathrm{M}$ MES buffer (Carl Roth: Art. No 4256.2) titrated to pH 4.8 with HCl and NaOH
- $0.1\,\mathrm{M}$ MES buffer diluted from the stock above
- $50\,\mathrm{mM}$ Borate buffer at pH 8.2 prepared by titrating boric acid (Carl Roth: Art. N0 6943.2) with HCl and NaOH
- 5'-amino-modified DNA diluted in deionized water to a concentration of $1\,\mathrm{mM}$

Ethyl-3-(3-dimethyl-aminopropyl) carbodiimide (EDC) (Merck: Art. No. E6383-1G) is stored in dry form in small aliquots of approximately $20\,\mathrm{mg}$ at $-20\,°\mathrm{C}$ and is later diluted in deionized water just before starting the bioconjugation reaction.

4.3. Colloid concentrations

Carboxylated silica spheres with diameters $1.01\,\mu\mathrm{m}$ (Lot: SiO_2−COOH-AR756-5ml) and $2.12\,\mu\mathrm{m}$ (Lot: SiO_2−COOH-AR1060-5ml) were purchased from the microParticles GmbH. The colloids come at a weight per volume concentration of $c_W = 0.05\,\mathrm{g/mL}$. This can be translated into a number density c via

$$c = \frac{c_W}{\rho \frac{4}{3}\pi \left(\frac{d}{2}\right)^3}$$

were $\rho = 1.85\,\mathrm{g/cm^3}$ is the density of the colloids and d is their diameter. The number density of the colloids is thus given by

$$c_{1.01} = \frac{50 \cdot 10^9}{\mathrm{mL}}$$
$$c_{2.12} = \frac{5.42 \cdot 10^9}{\mathrm{mL}}.$$

The following relation between the colloid concentrations c^s in the reaction has to be fulfilled in order to have approximately the same number of reaction sites in a sample:

$$c^s_{2.12} = \left(\frac{1.01}{2.12}\right)^2 c^s_{1.01}, \tag{8}$$

which relates the number densities via the surface area of the colloids. We choose

$$c^s_{1.01} = 10 \cdot 10^9 \frac{1}{mL}$$
$$c^s_{2.12} = 2.27 \cdot 10^9 \frac{1}{mL}.$$

as the starting point for our protocol.

4.4. Protocol

0. Colloid start concentration

- vortex colloid stock solutions
- 2.12 µm colloids: Pipet 419 µL of colloids and 581 µL of deionized water into a 1.5 mL tube (dilution 1/2.386)
- 1.01 µm colloids: Pipet 200 µL of colloids and 800 µL of deionized water into a 1.5 mL tube (dilution 1/5)

1. Transfer Colloids into MES buffer

- centrifuge the colloids at 250 rcf for 30 s and remove the supernatant (the Colloids should be sedimented)
- add 1 mL of 0.1 M MES buffer
- vortex briefly
- centrifuge the colloids at 250 rcf for 30 s (Colloids should be sedimented)
- add 100 µL of 0.1 M MES buffer at pH 4.8
- sonicate the colloids with a ultrasound generator at maximum power settings (20 W) for 1 min

The sonication with the ultrasound generator (Bandelin: SONOPLUS UW mini20) breaks up potentially aggregated colloids.

2. Modification reaction

- create 1.25 M EDC stock solution: Disolve 12 mg of EDC in 50 µL deionized water
- add 50 µL of 1 mM amino-modified DNA and 10 µL of 1 M MES-buffer and 40 µL of 1.25 M EDC to a 0.5 mL tube
- vortex briefly
- add the 100 µL of the prepared colloids solution to the sample.
- vortex briefly
- incubate for 3 h on a rotator

3. Washing procedure

The colloids are first washed with borate buffer to neutralize the acidic MES buffer and to hydrolize unreacted activated carboxyl groups. The colloids are then washed with deionized water to remove the borate buffer and any reactants that are unspecifically adsorbed to the colloids.

- transfer the sample to a 1.5 mL tube
- add 800 µL of 50 mM borate buffer to the sample.
- repeat the following steps for 5 times
  - centrifuge at 250 rcf for 30 s (the colloids should be sedimented) and remove the supernatant
  - add 1 mL of 50 mM borate buffer to the sample
  - vortex briefly; In round 4: sonicate at max settings for 1 min and incubate for 1.5 h on a rotator
- repeat the following steps for 5 times
  - Centrifuge at 1000 rcf for 30 s (Colloids should be sedimented) and remove the supernatant
  - add 1 mL of deionized water to the sample

  - vortex briefly; in round 4: sonicate at max setting for 1 min and incubate for 1.5 h on a rotator

- sonicate the colloids with a ultrasound generator at max settings for 1 min
- create aliquots, shock freeze in liquid nitrogen and store at $-80\,^{\circ}$C

5. RAW DATA

5.1. Buffer characterization

Supplementary Tables 1, 3, 5 and 7 list all velocity measurements for all buffer conditions. Column 1 and 2 list the concentrations of the buffer ingredients. Column 3 (video no.) enumerates the videos recorded with the same buffer condition. Column 4 (dimer (µm/s)) reports the measured velocities of all dimers in a video as an ordered list. Column 5 (orientation) lists the orientations of the dimers from Column 4 encoded as 1 ('up') and -1 ('down'). Up corresponds to the case where the smaller particle of a dimer appears below the larger particle on the computer screen. Column 6 (reference (µm/s)) contains the measured velocities of all reference particles as an ordered list. Column 7 (average reference (µm/s)) lists the average velocity of the reference particles from Column 6 of one video. Column 8 (dimer corrected (µm/s)) lists the dimer velocities from Column 4 corrected by the average velocity of the reference particles from Column 7.
Supplementary Tables 2, 4, 6 and 8 list the corrected velocity measurements, and the corresponding average and standard deviation for the given buffer compositions. Column 1 and 2 list the concentrations of the buffer ingredients. Column 3 (dimer corrected (µm/s)) list all corrected dimer velocities of a given buffer composition. Column 4 (average (µm/s)) reports the average of the corrected dimer velocities. Column 5 (standard deviation (µm/s)) lists the standard deviation of the corrected dimer velocities.

5.2. Electric field strength and frequency characterization

Table 9, 10 and 11 each contain data obtained from measurements made on one individual dimer and a reference particle. Column 1 and 2 list the applied electric field amplitude and

Supplementary Table 1. NaOH raw data

NaOH (μM)	$MgCl_2$ (μM)	video no.	dimer (μm/s)	orientation	reference (μm/s)	average reference (μm/s)	dimer corrected (μm/s)
10	5.2	1	-2.1584; 2.0315; 1.7996	1; -1; -1	-0.11963	-0.11963	2.0388; 2.1512; 1.9192
10	5.2	2	-2.11; 1.8049	1; -1	0.09221	0.09221	2.2022; 1.7127
10	5.2	3	1.7057; 1.6759	-1; -1	-0.22243; -0.26056; -0.17912	-0.2207	1.9264; 1.8966
25	0.2	1	-0.62871; -0.66528	-1; -1	-0.16556; -0.23086; -0.21944	-0.20529	-0.42343; -0.45999
25	0.2	2	-0.22598	1	-0.33294; -0.11588	-0.22441	0.0015664
25	0.2	3	-0.32598	-1	-0.1046	-0.1046	-0.22138
25	5.2	1	3.081; 2.9794	-1; -1	0.016034; 0.10796; 0.10584	0.076612	3.0044; 2.9028
25	5.2	2	-3.098; 3.1878	1; -1	-0.033192	-0.033192	3.0648; 3.221
25	5.2	3	-3.8004; 3.4204	1; -1	-0.24992; -0.36162; -0.092779; 0.1997	-0.12616	3.6742; 3.5466
50	0.2	1	-0.69124; 1.0838; 0.81608	-1; 1; 1	0.15563	0.15563	-0.84687; -0.92813; -0.66045
50	0.2	2	-1.0354; -1.1525	-1; -1	0.065881	0.065881	-1.1013; -1.2184
50	0.2	3	-0.69959; -0.90219; -0.88047	-1; -1; -1	-0.12209	-0.12209	-0.57749; -0.78009; -0.75838
50	5.2	1	0.36514; -0.75861	-1; 1	-0.011512	-0.011512	0.37665; 0.7471
50	5.2	2	0.49612; -0.9068	-1; 1	-0.12991; 0.10312; 0.039071	0.0040941	0.49203; 0.9109
50	5.2	3	-0.71939; -0.87973	1; 1	-0.09121	-0.09121	0.62818; 0.78852
100	5.2	1	-0.31532; -0.17464	1; -1	0.049767	0.049767	0.36509; -0.22441
100	5.2	2	0.05744; -0.03439	1; 1	-0.16706; 0.050236; -0.1582	-0.091673	-0.14911; -0.057283
100	5.2	3	-0.044189; 0.29934	1; 1	0.01613; -0.020345; -0.1041	-0.036106	0.0080835; -0.33544

Supplementary Table 2. NaOH corrected velocities

NaOH (μM)	$MgCl_2$ (μM)	dimer corrected (μm/s)	average (μm/s)	standard deviation (μm/s)
10	5.2	2.0388; 2.1512; 1.9192; 2.2022; 1.7127; 1.9264; 1.8966	1.9782	0.16687
25	0.2	-0.42343; -0.45999; 0.0015664; -0.22138	-0.27581	0.21261
25	5.2	3.0044; 2.9028; 3.0648; 3.221; 3.6742; 3.5466	3.2356	0.31069
50	0.2	-0.84687; -0.92813; -0.66045; -1.1013; -1.2184; -0.57749; -0.78009; -0.75838	-0.85889	0.2164
50	5.2	0.37665; 0.7471; 0.49203; 0.9109; 0.62818; 0.78852	0.65723	0.19825
100	5.2	0.36509; -0.22441; -0.14911; -0.057283; 0.0080835; -0.33544	-0.065514	0.24331

Supplementary Table 3. Tris raw data

Tris (μM)	$MgCl_2$ (μM)	video no.	dimer (μm/s)	orientation	reference (μm/s)	average reference (μm/s)	dimer corrected (μm/s)
10	5.2	1	2.1867; 2.112	-1; -1	-0.011658	-0.011658	2.1983; 2.1237
10	5.2	2	2.3178	-1	-0.28296; -0.093157	-0.18806	2.5058
10	5.2	3	2.0876; -2.5115	-1; 1	-0.01149	-0.01149	2.099; 2.5
10	5.2	4	2.384; -2.5342	-1; 1	-0.31553; 0.031664	-0.14193	2.526; 2.3922
25	5.2	1	2.7714; -1.5856	-1; 1	0.035405	0.035405	2.736; 1.621
25	5.2	2	-2.8863; 2.7368	1; -1	0.0074493	0.0074493	2.8938; 2.7293
25	5.2	3	-2.1166; -2.9565; 2.956	1; 1; -1	-0.0031657	-0.0031657	2.1135; 2.9533; 2.9592
50	0.2	1	0.24035; -0.29358; -0.29227	-1; 1; 1	-0.07472	-0.07472	0.31507; 0.21886; 0.21755
50	0.2	2	-0.18728; 0.3081; 0.067422	1; -1; -1	-0.10733; 0.014156	-0.046585	0.1407; 0.35468; 0.11401
50	0.2	3	-0.41175; -0.35746	1; 1	0.081775	0.081775	0.49352; 0.43924
50	5.2	1	2.2018; -2.4064	-1; 1	-0.082894	-0.082894	2.2847; 2.3235
50	5.2	2	2.2391; -2.415	-1; 1	0.042677; -0.078361	-0.017842	2.257; 2.3971
50	5.2	3	2.4588; -2.2549	-1; 1	-0.04447	-0.04447	2.5033; 2.2105
100	5.2	1	1.1616; -1.736; -1.8208	-1; 1; 1	-0.22214; -0.30256	-0.26235	1.424; 1.4736; 1.5584
100	5.2	2	1.7564; -1.4701	-1; 1	0.083002; 0.033984	0.058493	1.6979; 1.5286
100	5.2	3	-1.2916; 1.5386; -1.7132	1; -1; 1	-0.071143; 0.016444	-0.027349	1.2642; 1.566; 1.6859
250	5.2	1	0.82139; -0.63782	-1; 1	-0.016064	-0.016064	0.83745; 0.62176
250	5.2	2	-0.87382; -0.94419	1; 1	-0.03497	-0.03497	0.83885; 0.90922
250	5.2	3	-0.95719; -0.82211; 0.80946	1; 1; -1	-0.13714; 0.20802	0.035439	0.99263; 0.85755; 0.77402
500	5.2	1	-0.55723; 0.51097; -0.45549	1; -1; 1	-0.065543	-0.065543	0.49169; 0.57651; 0.38994
500	5.2	3	-0.54143; 0.78223	1; -1	-0.2473	-0.2473	0.29413; 1.0295

Supplementary Table 4. Tris corrected velocities

Tris (μM)	$MgCl_2$ (μM)	dimer corrected (μm/s)	average (μm/s)	standard deviation (μm/s)
10	5.2	2.1983; 2.1237; 2.5058; 2.099; 2.5; 2.526; 2.3922	2.335	0.18938
25	5.2	2.736; 1.621; 2.8938; 2.7293; 2.1135; 2.9533; 2.9592	2.5723	0.51075
50	0.2	0.31507; 0.21886; 0.21755; 0.1407; 0.35468; 0.11401; 0.49352; 0.43924	0.2867	0.13732
50	5.2	2.2847; 2.3235; 2.257; 2.3971; 2.5033; 2.2105	2.3293	0.10607
100	5.2	1.424; 1.4736; 1.5584; 1.6979; 1.5286; 1.2642; 1.566; 1.6859	1.5248	0.14114
250	5.2	0.83745; 0.62176; 0.83885; 0.90922; 0.99263; 0.85755; 0.77402	0.83307	0.11551
500	5.2	0.49169; 0.57651; 0.38994; 0.29413; 1.0295	0.55636	0.28501

Supplementary Table 5. NaCl raw data

NaCl (μM)	$MgCl_2$ (μM)	video no.	dimer (μm/s)	orientation	reference (μm/s)	average reference (μm/s)	dimer corrected (μm/s)
25	5.2	1	0.2565; -0.090399	-1; -1	-0.047559; -0.10738	-0.077468	0.33397; -0.01293
25	5.2	2	-0.0010654; -0.072518	1; -1	-0.083108	-0.083108	-0.082042; 0.01059
25	5.2	3	-0.058622; 0.077811; 0.20893	1; -1; -1	-0.0033616	-0.0033616	0.055261; 0.081173; 0.21229
50	0.2	1	-0.07064	-1	-0.10322	-0.10322	0.032583
50	0.2	2	-0.30739; 0.14427	-1; 1	-0.20242	-0.20242	-0.10497; -0.34669
50	0.2	3	0.23731; -0.0034925	1; 1	0.022871; 0.14171	0.08229	-0.15502; 0.085783
50	5.2	1	0.18548; -0.60578	-1; 1	0.10408; -0.16269; -0.13499	-0.064532	0.25002; 0.54125
50	5.2	2	0.35203; -0.4395	-1; 1	0.013808; 0.12143	0.067621	0.28441; 0.50712
50	5.2	3	-0.36842; 0.49881	1; -1	0.041369; -0.13753	-0.048079	0.32034; 0.54689
50	5.2	4	-0.62647; 0.5147; -0.6237	1; -1; 1	-0.011805; 0.0073405	-0.0022323	0.62424; 0.51693; 0.62146
100	0.2	1	-0.3359; -0.070846; -0.32053	1; 1; 1	-0.39082; -0.018323	-0.20457	0.13133; -0.13372; 0.11596
100	0.2	2	-0.050905	-1	-0.15048; -0.098426; -0.10022; 0.21803; -0.13712	-0.053645	0.0027406
100	0.2	3	0.041658; 0.27696; 0.091807	-1; -1; -1	0.035803; 0.092218	0.06401	-0.022352; 0.21295; 0.027797
100	0.2	4	-0.19775	1	-0.035193; 0.050215; -0.069265	-0.018081	0.17967
100	5.2	1	0.54022; -0.76891	-1; 1	-0.064309	-0.064309	0.60453; 0.7046
100	5.2	2	-0.9319; -0.94887	1; 1	-0.061837; 0.0075117; -0.099579	-0.051301	0.88059; 0.89757
100	5.2	3	0.51296; 0.58286	-1; -1	0.1632	0.1632	0.34976; 0.41966
100	5.2	4	0.59116; 0.54284	-1; -1	-0.0032619	-0.0032619	0.59442; 0.5461
250	5.2	1	0.35226; -0.81461	-1; 1	0.10234	0.10234	0.24992; 0.91694
250	5.2	2	0.57366; 0.52717; -0.74718	-1; -1; 1	-0.012219	-0.012219	0.58588; 0.53939; 0.73496
250	5.2	3	-0.43561; 0.52548	1; -1	-0.007625	-0.007625	0.42798; 0.53311
500	5.2	1	-0.23991; 0.53692; -0.27462	1; -1; 1	0.118; -0.19101	-0.036507	0.20341; 0.57343; 0.23812
500	5.2	2	0.69386; -0.13413	-1; 1	-0.16074	-0.16074	0.8546; -0.026605
500	5.2	3	0.36688; 0.44444	-1; -1	-0.10812	-0.10812	0.475; 0.55257

frequency. Column 3 lists the measured velocity of the dimer. Column 4 lists the velocity of the reference particle. Column 5 lists the corrected velocity of the dimer.

Supplementary Table 6. NaCl corrected velocities

NaCl (μM)	$MgCl_2$ (μM)	dimer corrected (μm/s)	average (μm/s)	standard deviation (μm/s)
25	5.2	0.33397; -0.01293; -0.082042; 0.01059; 0.055261; 0.081173; 0.21229	0.085472	0.14266
50	0.2	0.032583; -0.10497; -0.34669; -0.15502; 0.085783	-0.097663	0.17027
50	5.2	0.25002; 0.54125; 0.28441; 0.50712; 0.32034; 0.54689; 0.62424; 0.51693; 0.62146	0.46807	0.14427
100	0.2	0.13133; -0.13372; 0.11596; 0.0027406; -0.022352; 0.21295; 0.027797; 0.17967	0.064296	0.11614
100	5.2	0.60453; 0.7046; 0.88059; 0.89757; 0.34976; 0.41966; 0.59442; 0.5461	0.62465	0.19693
250	5.2	0.24992; 0.91694; 0.58588; 0.53939; 0.73496; 0.42798; 0.53311	0.56974	0.21321
500	5.2	0.20341; 0.57343; 0.23812; 0.8546; -0.026605; 0.475; 0.55257	0.41007	0.29205

Supplementary Table 7. $MgCl_2$ raw data

$MgCl_2$ (μM)	video no.	dimer (μm/s)	orientation	reference (μm/s)	average reference (μm/s)	dimer corrected (μm/s)
5	4	-0.1007	1	0.12329; 0.078374; 0.08571	0.095791	0.19649
5	1	0.89903; -0.73302	1; -1	0.033914	0.033914	-0.86511; -0.76694
5	2	0.50023; -0.76023	1; -1	0.040048	0.040048	-0.46019; -0.80028
5	3	0.61077; 0.64962	1; 1	-0.071589	-0.071589	-0.68236; -0.72121
10	1	-0.77615	-1	-0.11222	-0.11222	-0.66393
10	2	0.81232	1	-0.049807; 0.027225; -0.089389; 0.061394	-0.012644	-0.82496
10	3	-0.67114; -0.61298	-1; -1	0.16781; 0.0054852; -0.045543; 0.050324	0.04452	-0.71566; -0.6575
10	4	0.94059	1	-0.1349	-0.1349	-1.0755
10	5	-0.44429	-1	-0.061833; 0.035126; 0.065325	0.012873	-0.45717
25	1	-0.2533; 0.49167	1; 1	-0.20812	-0.20812	0.045177; -0.69979
25	2	-0.68736; -0.14944	-1; -1	-0.041324; -0.064957; -0.036963	-0.047748	-0.63961; -0.10169
25	3	-0.40842	-1	-0.099255	-0.099255	-0.30917
25	4	-0.31028; 0.63511	-1; 1	-0.021058	-0.021058	-0.28922; -0.65617
50	1	-0.043009	-1	0.0075411	0.0075411	-0.05055
50	2	-0.1422; -0.13616	-1; 1	0.0054712	0.0054712	-0.14768; 0.14163
50	3	-0.079519; 0.080918	-1; 1	-0.045867	-0.045867	-0.033653; -0.12679
100	1	0.11921	-1	0.0010629	0.0010629	0.11815
100	2	0.18407	-1	0.074598; 0.051411	0.063005	0.12106
100	3	0.31911; 0.23815	-1; -1	-0.0078792; -0.064006	-0.035943	0.35505; 0.27409
100	4	-0.46523	1	0.066031; 0.24479	0.15541	0.62064
100	5	0.29586; -0.21409	-1; 1	-0.060438	-0.060438	0.35629; 0.15366
250	1	-0.2334	1	-0.062887	-0.062887	0.17051
250	2	0.24637	-1	0.0055673; -0.030459; -0.0033249	-0.0094054	0.25577
250	3	0.33192; -0.28766	-1; 1	0.061578	0.061578	0.27034; 0.34924

Supplementary Table 8. $MgCl_2$ corrected velocities

$MgCl_2$ (μM)	dimer corrected (μm/s)	average (μm/s)	standard deviation (μm/s)
5	-0.86511; -0.76694; -0.46019; -0.80028; -0.68236; -0.72121	-0.71601	0.14038
10	-0.66393; -0.82496; -0.71566; -0.6575; -1.0755; -0.45717	-0.73245	0.20621
25	0.045177; -0.69979; -0.63961; -0.10169; -0.30917; -0.28922; -0.65617	-0.37864	0.29366
50	-0.05055; -0.14768; 0.14163; -0.033653; -0.12679; 0.19649	-0.0034233	0.14154
100	0.11815; 0.12106; 0.35505; 0.27409; 0.62064; 0.35629; 0.15366	0.28556	0.18015
250	0.17051; 0.25577; 0.27034; 0.34924	0.26147	0.073229

Supplementary Table 9. Swimmer 1

electric field (mV/μm)	frequency (Hz)	dimer (μm/s)	reference (μm/s)	dimer corrected (μm/s)
3.05	250	-0.04425	-0.05515	0.01089
4.57	250	0.18415	-0.07716	0.26130
6.09	250	-0.36709	-0.05731	0.30978
7.61	250	-0.58703	-0.06520	0.52183
9.14	250	0.73478	-0.10905	0.84382
10.66	250	-1.01412	-0.04195	0.97218
12.18	250	1.08429	-0.07286	1.15715
13.71	250	-1.58886	-0.00330	1.58556
15.23	250	1.90124	0.06676	1.83448
16.75	250	-2.27900	0.02538	2.30438
16.75	500	-1.90459	0.10462	2.00922
16.75	750	1.27442	0.04976	1.22466
16.75	1000	0.84186	-0.06135	0.90321
16.75	1500	-0.14115	0.23458	0.37573
16.75	2000	0.00711	0.11272	0.10561

Supplementary Table 10. Swimmer 2

electric field (mV/µm)	frequency (Hz)	dimer (µm/s)	reference (µm/s)	dimer corrected (µm/s)
3.05	250	-0.07672	-0.03346	0.04326
4.57	250	-0.23488	-0.05509	0.17980
6.09	250	0.29167	-0.13350	0.42517
7.61	250	0.50567	-0.10702	0.61269
9.14	250	-0.96007	-0.15874	0.80133
10.66	250	0.91369	-0.09650	1.01020
12.18	250	1.37226	-0.08227	1.45453
13.71	250	-1.85958	-0.19601	1.66357
15.23	250	2.01217	-0.13712	2.14930
16.75	250	-2.41953	0.00508	2.42461
16.75	500	1.90613	-0.27114	2.17727
16.75	750	-1.64252	-0.34347	1.29905
16.75	1000	-1.02669	-0.22037	0.80632
16.75	1500	-0.51470	-0.02538	0.48932
16.75	2000	-0.40936	-0.36060	0.04876

Supplementary Table 11. Swimmer 3

electric field (mV/µm)	frequency (Hz)	dimer (µm/s)	reference (µm/s)	dimer corrected (µm/s)
3.05	250	-0.05914	0.00575	0.06489
4.57	250	0.13688	-0.03549	0.17237
6.09	250	0.16393	-0.06861	0.23254
7.61	250	0.37440	-0.14963	0.52403
9.14	250	0.53618	-0.12732	0.66351
10.66	250	-1.00302	-0.16564	0.83737
12.18	250	-1.13091	0.05384	1.18474
13.71	250	1.15126	-0.26317	1.41444
15.23	250	1.65991	-0.01931	1.67923
16.75	250	-1.95483	-0.12504	1.82979
16.75	500	-1.72144	-0.12492	1.59652
16.75	750	-1.33342	-0.13304	1.20038
16.75	1000	0.71604	-0.08498	0.80102
16.75	1500	-0.41806	-0.11272	0.30534
16.75	2000	0.17822	0.09728	0.08093

[1] Fernández-Mateo, R., García-Sánchez, P., Calero, V., Morgan, H. & Ramos, A. Stationary electro-osmotic flow driven by ac fields around charged dielectric spheres. *Journal of Fluid Mechanics* **924**, R2 (2021).

[2] Khair, A. S. & Balu, B. Breaking electrolyte symmetry in induced-charge electro-osmosis. *Journal of Fluid Mechanics* **905**, A20 (2020).

[3] Squires, T. M. & Bazant, M. Z. Induced-charge electro-osmosis. *Journal of Fluid Mechanics* **509**, 217–252 (2004).

[4] Schnitzer, O. & Yariv, E. Strong electro-osmotic flows about dielectric surfaces of zero surface charge. *Physical review. E, Statistical, nonlinear, and soft matter physics* **89**, 043005 (2014).

[5] Tseng, Q. *et al.* A new micropatterning method of soft substrates reveals that different tumorigenic signals can promote or reduce cell contraction levels. *Lab on a chip* **11**, 2231–2240 (2011). URL https://pubmed.ncbi.nlm.nih.gov/21523273/.
