# Supplementary material for: Microrobots powered by concentration polarization electrophoresis (CPEP)
Source: Nat Commun. 2023 Oct 6;14:6247. doi: 10.1038/s41467-023-41923-1 (PMC10558450; doi:10.1038/s41467-023-41923-1)
Supplement: Supplementary file 3 — Description of Additional Supplementary Files [file 41467_2023_41923_MOESM3_ESM.docx]

**File Name:** Supplementary Movie 1.mp4

**Description:**

Collection of microscopy videos to demonstrate the function and applications of our micro swimmers. The videos where used to create Fig.1-4.

**File Name:** Supplementary Movie 2.mp4

**Description:**

Collection of microscopy videos that show various types of fragmented particles that exhibit directed propulsion in an AC electric field. The part labelled ‘Brewers Yeast’ was used to create Fig.7.

**File Name:** Supplementary Movie 3.mp4

**Description:**

Collection of microscopy videos that show a forwards moving micro swimmer passing by tracer particles. The video was edited such that the micro swimmer appears stationary. The video was used to create Supplementary Fig.5a.

**File Name:** Supplementary Movie 4.mp4

**Description:**

Collection of microscopy videos that show a backwards moving micro swimmer passing by tracer particles. The video was edited such that the micro swimmer appears stationary. The video was used to create Supplementary Fig.5b.
